# Gastrocnemius–soleus intramuscular lipoma misinterpreted as hemangioma: a rare case report

**DOI:** 10.1097/MS9.0000000000004070

**Published:** 2025-10-13

**Authors:** Mandeep D. Joshi, Nishant Pandey, Kenedy Khatri, Avinash Kc, Ram Thapa

**Affiliations:** a Department of Dermatology, Manipal College of Medical Sciences, Kaski, Nepal; b Manipal College of Medical Sciences, Kaski, Nepal; c Department of Orthopedics, Green Pastures Hospital, Pokhara, Kaski, Nepal; d Department of Pathology, Manipal College of Medical Sciences, Kaski, Nepal

**Keywords:** case report, hemangioma, intramuscular lipoma

## Abstract

**Introduction:**

Intramuscular lipomas (IMLs) are rare, comprising less than 1% of all lipomas, and typically arise in large proximal muscles. Their occurrence in the calf is extremely uncommon and may mimic vascular or malignant lesions on imaging.

**Case Presentation:**

A 45-year-old female presented with an 8-month history of dull pain in the left calf. MRI suggested a hemangioma. Surgical excision was performed, and histopathology confirmed an intramuscular lipoma. The patient recovered without complications and reported full symptom resolution.

**Clinical Discussion:**

IMLs in distal sites like the calf are rare and challenging to diagnose due to imaging overlap with hemangiomas and liposarcomas. MRI aids evaluation, but histopathology remains the gold standard. Surgical excision is curative, and biopsy may be omitted in select small, radiologically benign lesions to avoid contamination.

**Conclusion:**

IMLs should be considered in the differential diagnosis of painful intramuscular masses, even in atypical locations. Accurate diagnosis and complete excision ensure favorable outcomes.

## Introduction

Lipomas are the most common benign mesenchymal tumors, generally composed of mature adipocytes and found in the subcutaneous tissue^[1]^. They can also occur in deeper locations such as intermuscular, intramuscular, intraosseous, periosteal, and visceral sites^[2]^. Intramuscular lipomas (IMLs) are a rare subtype, representing less than 1% of all lipomas and about 1.8% of primary adipose tissue tumors^[3]^. They arise within muscle fibers and are further classified histologically into infiltrative (83%) and well-circumscribed (17%) types^[4]^. IMLs commonly affect large muscles of the trunk and proximal limbs, especially the thigh, shoulder, and upper arm, and are rare in distal regions like the hand and foot^[1,4]^. These lesions typically occur between the ages of 40–70 years but can be seen at any age^[3]^. Clinically, histologically, and radiologically, they may mimic well-differentiated liposarcomas, complicating diagnosis^[3]^. Treatment is primarily surgical – marginal excision for well-defined types and wide excision for infiltrative ones^[4]^. Minimally invasive endoscopic approaches have been reported in select cases for improved cosmetic outcomes^[4]^. This case report has been reported in line with the SCARE checklist^[5]^.

**HIGHLIGHTS**

- Intramuscular lipomas (IMLs) of the calf are rare and can mimic vascular or malignant lesions on imaging.

- MRI may suggest hemangioma due to serpiginous vessels and postcontrast enhancement, leading to potential misdiagnosis.
- Histopathology remains the gold standard for definitive diagnosis, distinguishing IML from liposarcoma or hemangioma.
- Complete surgical excision ensures symptom resolution and minimizes recurrence risk.
- In selected small, benign-appearing lesions, biopsy-excision may be preferred over preoperative biopsy to reduce contamination risk.

## Case presentation

### Timeline

A 45-year-old female presented with an 8-month history of dull, aching pain over the posterior aspect of the left calf. There was no history of trauma, fever, weight loss, or other constitutional symptoms. The pain was insidious in onset and gradually progressive. On clinical examination, a well-defined, palpable lump was noted in the medial calf region. The mass was smooth, nonpulsatile, mildly tender, and not fixed to overlying skin. No regional lymphadenopathy was present, and neurological examination of the limb revealed no deficits.

Routine laboratory investigations were mostly within normal limits: random blood sugar, urea, creatinine, serum sodium and potassium, thyroid stimulating hormone, renal function tests, bleeding time, total count, differential count, red blood cell count, and white blood cell count were all normal. Hemoglobin was slightly low (11.6 g/dL), and the platelet count was slightly elevated at 306 000 cells/cm³. Prothrombin time and international normalized ratio were within normal range. Screening for HIV-1 and 2, HBsAg, and HCV was nonreactive. Electrocardiogram was also normal.

Plain radiographs of the leg were unremarkable. MRI of the left leg was performed using 3T-CE protocol, including axial T2, sagittal T1 and T2, coronal T1 and T2, and fat-suppressed sequences. Imaging revealed a serpiginous cluster of vessels with fatty tissue in the superficial aspect of the proximal gastrocnemius lateralis muscle, mildly extending into the adjacent soleus muscle laterally. Mild heterogeneous postcontrast enhancement was observed, with a few draining branches into the short saphenous vein. The tibia and fibula demonstrated normal signal intensities, with no lytic or sclerotic lesions or periosteal reaction (Fig. 2A and 2B). The remaining muscles, joints, vessels, and nerves were normal. No joint effusion was present. These imaging findings, including a well-defined vascular channel associated with fatty tissue, mild heterogeneous enhancement, absence of thick or nodular septa, necrosis, or nonadipose nodules, and stable surrounding bone and muscle signal intensities, strongly suggested a benign lesion, consistent with a hemangioma, venous malformation, or an intramuscular lipoma. Vascular channels and draining veins were consistent with hemangioma, despite the abundant fatty component present in the tumor. The calf muscles, which are highly vascularized, further added to our confusion. However, based on clinical presentation and imaging findings, an intramuscular lipoma was considered most likely.

**Figure 1.**
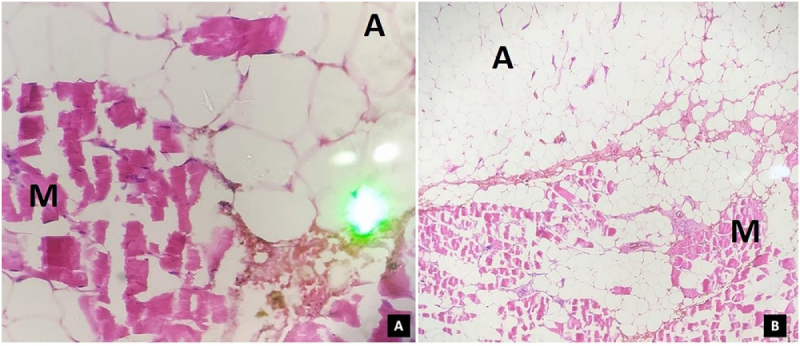
(A) Histological section stained with hematoxylin and eosin (H&E) at 40× magnification showing mature adipose tissue infiltrating between skeletal muscle fibers. The adipocytes exhibit abundant, clear cytoplasm with peripherally pushed nuclei. Fibrovascular septa are also visible within the infiltrated areas. (B) H&E-stained section at 10× magnification revealing lobules of mature adipose tissue diffusely infiltrating skeletal muscle, along with thin- and thick-walled vascular channels and areas of hemorrhage. M: muscle tissue; A: adipose tissue.

Surgical excision was planned for definitive diagnosis and symptomatic relief. Grossly, the specimen measured 6 × 5 × 3 cm and consisted of soft, pale white to grey-brown nodular tissue (Table 1). The cut surface showed lobules of adipose tissue interspersed with muscle. Histopathology revealed a partially encapsulated mass composed of mature adipose tissue infiltrating between skeletal muscle fibers, separated by fibrovascular septa with thin and thick-walled vascular channels and areas of hemorrhage, without evidence of atypia or mitosis. A final diagnosis of intramuscular lipoma was made (Fig. 1A, B). The patient had an uneventful postoperative recovery. At the time of discharge, the wound edge was dry with no redness or signs of infection. She was discharged with advice for 14 days of bed rest, leg elevation, and a short course of oral medications. On 1-month and 3-month follow-ups in out-patient settings, the patient was doing well without residual pain, with a well-healed surgical site, and no clinical evidence of local recurrence. No further imaging was deemed necessary on grounds of clinical examination. On 6-month follow-up via telephone, there was no recurrence of the lesion.

**Figure 2.**
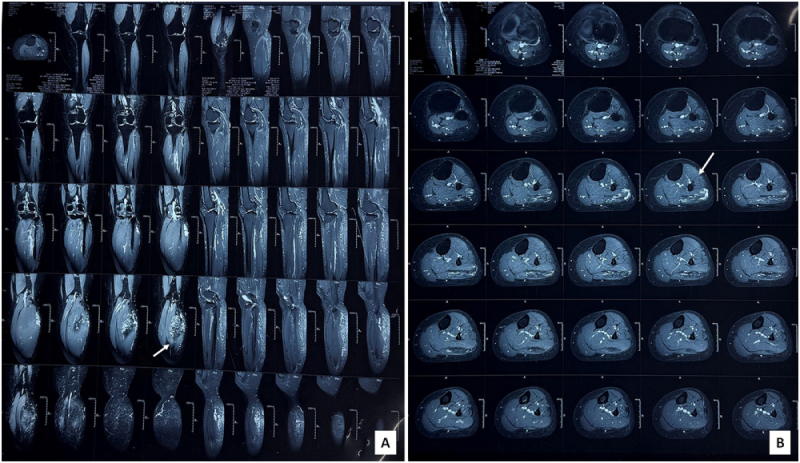
(A) Coronal and sagittal MRI images showing a serpiginous cluster of vessels with fatty tissue in the superficial aspect of the proximal gastrocnemius lateralis muscle (arrow), with mild extension into the adjacent soleus muscle. (B) Axial T2-weighted MRI image demonstrating the vascular lesion with heterogeneous signal intensity and draining branches into the short saphenous vein (arrow).

**Table 1:** Timeline of the events

Time period	Events
Month 0	Symptom onset, dull aching calf pain, and insidious onset
Months 1–7	Progressive pain, palpable mass development
Month 8	Clinical examination, palpable tender mass, and normal neurology
	Laboratory tests are normal and plain X-rays are unremarkable
	MRI performed, serpiginous vessels, fatty tissue, and hemangioma suspected. Surgical excision, 6 × 5 × 3 cm specimen. Mature adipose tissue, muscle infiltration, and intramuscular lipoma confirmed
Postoperative/follow-up	Uneventful recovery and complete symptom resolution

## Discussion

IMLs are rare benign tumors, accounting for less than 1% of all lipomas. They typically occur within large muscles of the limbs and trunk, with the thigh being the most frequently involved site, followed by the shoulder and upper arm. Most documented cases involve the upper limb or chest wall, making the calf a rare location for these tumors^[1,3]^. This anatomical specificity emphasizes the importance of maintaining high clinical suspicion in patients with unexplained limb pain, even when the location is atypical. The diagnosis of IMLs can be challenging due to their clinical and radiological resemblance to other soft-tissue lesions such as hemangiomas and well-differentiated liposarcomas^[3,6]^. In particular, intramuscular hemangiomas may contain significant adipose components that mimic lipomas by replacing adjacent muscle^[3,7]^. Histologically, well-differentiated liposarcomas can be distinguished from IMLs by the presence of atypical cells or vacuolated lipoblasts within thick fibrous septa and admixed fibroblast-like spindle cells, along with more prominent vasculature^[3]^.

MRI plays a key role in the preoperative evaluation of these lesions, though it may not always offer definitive differentiation. Well-differentiated liposarcomas and IMLs may appear similar on imaging due to their shared lipomatous composition^[1]^. Well-differentiated liposarcoma are usually larger (>10 cm)^[8]^, with lower percentage fat (<75%), have thick or nodular septa (>2 mm), with nodular or globular areas, prominent high T2 signal foci, significant enhancement, associated nonadipose tissue component, and preferential location in the lower limb intramuscular component^[9–11]^. In our case, the MRI showed serpiginous vessels with fatty tissue and mild postcontrast enhancement, initially suggesting a hemangioma – a diagnostic pitfall highlighted in previous literature. Summary of the findings supporting and contradictory to intramuscular lipoma vs hemangioma in our case are highlighted in Table 2.

**Table 2:** Differential diagnosis: imaging features supporting and contradicting hemangioma vs intramuscular lipoma

Diagnosis	Supporting features	Contradictory features
Hemangioma	Presence of serpiginous vascular channels	Abundant fatty tissue component, mild enhancement patterns (hemangiomas usually demonstrate more avid enhancement)^[12]^
	Identifiable draining veins into short saphenous veins	
Intramuscular lipoma	Prominent fatty tissue composition	Presence of prominent vascular channels is uncommon in a typical lipoma^[3]^
	Well-defined margins^[13]^	
	Absence of thick or nodular septa and stable features^[9–11]^	
		Draining veins with postcontrast enhancement

McTighe *et al* noted that infiltrative IMLs may exhibit heterogeneous signals on imaging due to interdigitation of fat and muscle fibers^[3]^. Despite advancements in imaging, histopathological evaluation remains the gold standard for diagnosis. In our patient, the absence of atypia and lipoblasts confirmed a benign intramuscular lipoma^[3,7]^. While histology is the cornerstone for diagnosis, cytogenetic analysis is increasingly being integrated into the diagnostic workup for lipomatous tumors^[3]^. Importantly, the infiltrative pattern and intramuscular location of these tumors often require differentiation from soft-tissue sarcomas, with radiologic features supporting but not replacing histologic confirmation^[7]^. Surgical excision remains the definitive treatment for symptomatic IMLs. Marginal resection is recommended for well-circumscribed lesions, while wide excision is preferred for infiltrative types to minimize recurrence^[4,7]^. Our patient underwent complete excision without complications and experienced full symptom resolution – an outcome consistent with findings by Su *et al* and Ramos-Pascua *et al*, who reported low recurrence rates following total excision^[1,3,14,15]^. Generally accepted indications for surgery include tumors larger than 5 cm, deep-seated lesions in the proximal lower extremities, infiltrative growth, or recurrence after prior resection despite benign histology^[4,16]^. While our case had a favorable short-term outcome, the absence of long-term follow-up limits assessment of recurrence risk. Additionally, a preoperative biopsy might have helped clarify the diagnosis earlier. However, as Le Nail *et al* suggest, biopsy may be avoided in small (1 cm), radiologically benign tumors to prevent contamination of adjacent healthy tissue. In such cases, a biopsy-excision approach may be justified, provided safe and complete margins can be obtained^[17]^.

Our case highlights that IMLs, although uncommon, should be considered in the differential diagnosis of painful intramuscular masses, even when imaging suggests vascular lesions. Histopathology remains essential for definitive diagnosis, and complete surgical excision continues to be the cornerstone of effective management. Future research should focus on the role of advanced imaging modalities to improve diagnostic accuracy, particularly in atypical presentations. In this frontier, artificial intelligence (AI) may be one of the most promising areas of research. AI uses models that can be trained to discern the likelihood of benign vs malignant tumors, including tumors with overlapping imaging characteristics. The limitations of humans are well documented in literature. For instance, a study by Gaskin and Helms, which retrospectively reviewed 126 imaged fatty masses and compared MRI examinations with their prospective interpretations and corresponding pathology reports, demonstrated that MRI interpretation achieves 100% sensitivity but only 83% specificity for well-differentiated liposarcoma, with a positive predictive value of only 38% due to overlaps with benign lipoma variants^[10]^. Building on these findings, AI and radiomics have shown great promise in improving diagnostic accuracy by extracting detailed quantitative imaging features from MRI scans that go beyond visual assessment possible by radiologists alone. Models trained on these features can more reliably differentiate tumors, for instance, liposarcoma from lipoma with diagnostic accuracy upward of 90%^[18]^, and intramuscular lipoma with hemangioma, as in our case, thereby reducing false positives common in conventional MRI interpretation. AI models enhance differentiation by analyzing texture, shape, and intensity patterns, thus helping to overcome the specificity limitations observed in traditional MRI readings^[19]^. This demonstrates the significant potential of AI in diagnostic imaging, especially in cases like ours, where diagnostic dilemma is quite common.

## Conclusion

This case highlights the diagnostic challenge of atypically located IMLs and reinforces the importance of histopathological confirmation. Lack of long-term follow-up prevents assessment of recurrence risk. Intramuscular lipoma, though rare in the calf region, should be considered in the differential diagnosis of painful muscular swellings. Imaging may be misleading, and histopathological confirmation is crucial. Complete excision remains the cornerstone of effective management.

### Strength and limitations

This case highlights the diagnostic challenge of atypically located IMLs and reinforces the importance of histopathological confirmation.

## Data Availability

The data that support the findings of this study will be available from the corresponding author upon reasonable request.

## Research registration unique identifying number (UIN)

This case report does not have registration.
